# Quantifying National-Scale Changes in Agricultural Land Exposure to Fluvial Flooding

**DOI:** 10.3390/su132212495

**Published:** 2021

**Authors:** Heather Craig, Ryan Paulik, Utkur Djanibekov, Patrick Walsh, Alec Wild, Benjamin Popovich

**Affiliations:** 1 National Institute of Weather and Atmospheric Research (NIWA), Auckland 1010, New Zealand; 2 Manaaki Whenua-Landcare Research, 231 Morrin Road, St. Johns, Auckland 1072, New Zealand; 3 United States Environmental Protection Agency, 1200 Pennsylvania Ave. NW, Washington, DC 20460, USA; 4 Moffatt & Nichol, 1780 Hughes Landing Boulevard, Suite 575, The Woodlands, TX 77380, USA

**Keywords:** land use, exposure, flooding, agriculture, dairy farming, economic exposure, RiskScape

## Abstract

This study quantifies the exposure of agricultural land in Aotearoa-New Zealand’s (A-NZ) flood hazard zones (FHZs). We developed a spatio-temporal flood exposure framework to quantify the extent of the area and yearly earnings before income and tax (EBIT) for arable, forestry, horticulture, sheep and beef, and dairy land in FHZs between 1990 and 2016. In 1990, ~1.57 million hectares of agricultural land were exposed, decreasing slightly to ~1.50 million hectares by 2016. However, there was a change in the lower-value types of agricultural land uses being exposed, such as for sheep and beef farming and forestry, toward dairy farming (from ~364,000 hectares in FHZs in 2008 to ~471,000 hectares in 2016). Dairy farming is more intensively staffed with larger amounts of fixed assets, making them less resilient to flood impacts. Despite this, conversion to dairy farming even within the identified FHZs has been driven by the increasing profitability of the enterprise. As a result of both the production value change and land area increases, the dairy EBIT values within FHZs rose rapidly from NZD 382 million to NZD 1.25 billion between 2008 and 2012, creating significantly more economic exposure for A-NZ. This trend is particularly evident in the Southland, Canterbury, and Waikato regions. Similarly, in the Marlborough, Tasman, and Hawke’s Bay regions, there was an increase in high-value horticultural land—predominantly viticulture—in FHZs (a increase of NZD 321 million in annual EBIT for exposed horticulture across the three regions). Identifying sub-national trends in agricultural flood exposure allows for a detailed analysis of the likely impacts in high-risk areas, which can inform emergency management plans and mitigative actions that diminish the economic impacts from flood events.

## 1. Introduction

Flooding is the most frequent damaging natural hazard in Aotearoa-New Zealand (A-NZ) [1]. The growing exposure of buildings, infrastructure, and agriculture to flooding has led to increasing insurance claims [2]. Agricultural industries are particularly vulnerable to flooding, as they are often concentrated in fertile floodplains and require relatively large amounts of land compared to other industries [3]. They are also supplied by several interconnected industries (such as electricity networks and feed and fertilizer suppliers) that may sustain damage and/or disruption during flood events. The highly complex network of agricultural suppliers and customers leaves the industry highly vulnerable to adverse events and their cascading impacts [4]. Climate change will both increase the frequency and intensity of flood events and further reduce resilience for climate-sensitive industries as areas that were once highly productive become more marginal in their utility [5,6]. With this in mind, governments and policymakers are increasingly focusing on improving the ability of infrastructure and industry to recover their functions and structure after damaging flood events through resilience-building and vulnerability-reducing policy and planning measures [7].

Flood events have caused significant damage and economic losses to agricultural systems, with these losses continuing to increase with agricultural intensification and climate-change-induced increases in extreme events [8]. Significant agricultural production losses and capital asset damage have occurred due to past flood events in A-NZ and internationally. The 2017 Bay of Plenty floods resulted in agricultural production losses and capital asset damage with individual farm-scale losses of up to NZD 890,000 [9]. The 2004 Manawatu floods also caused significant estimated costs to dairy farming (NZD 41.4 million), pastoral land (NZD 66 million), arable and cropping land (NZD 24 million), and forestry farming (NZD 29 million). Globally, in 2012, persistent heavy rainfall caused losses of over NZD 1700 per hectare to grassland agriculture in Somerset, England, leading to significant insurance claims [10]. Further studies show that for the Northwest region of England, 7% of arable agricultural land will become unproductive due to frequent flooding in the next 100 years [11]. In Vietnam, exposure modeling shows that for 10%, 5%, and 1% annual exceedance probability (AEP) flood events, 27%, 31%, and 33% of agricultural land would be inundated nationally. This will mostly impact rice crops and is of particular concern with the frequency of these events increasing substantially in Southeast Asia [12]. Food and economic security is also threatened by frequent flooding of agricultural areas in Borneo, Indonesia, where over 700 rural settlements reported flooding in three years (2013–2016) [13]. The increasing incidence of damaging flooding is having catastrophic socio-economic effects in countries such as Nigeria, where agricultural losses are becoming a yearly event, resulting in the of curtailing food availability and the ability to undertake agricultural trade in a region where poverty is widespread [14]. Similar increases in the occurrence and severity of flooding, the exposure and vulnerability of agricultural land and associated assets, and the resulting financial and economic losses have been observed in a diverse range of countries and socio-economic settings, including China [15], Pakistan [16], the United Kingdom [17,18], Czech Republic [19], Finland [20], the United States [21-23], Australia [24], and A-NZ [3,9].

The increasing incidence of exposure of agricultural land to flooding heightens the need to investigate industrial vulnerabilities in response to climate and socio-economic change [25]. Previous research focused on quantifying direct economic losses has had a small spatial scope (e.g., floodplains) or has been specific to an industry, thus preventing analyses of national- or regional-scale exposure of agricultural industries to flooding [12,26-28]. The detection and monitoring of large-scale trends in exposure of agricultural industries to flooding are essential for implementing national and regional social and economic policies and strategies that enable industries and farmers to prosper under present and future climate conditions.

This study investigates agricultural exposure to flooding in A-NZ over a 26-year period between 1990 and 2016. A spatio-temporal flood exposure framework was developed by using the RiskScape software engine to quantify the extent of the area and yearly earnings before income and tax (EBIT) for arable, forestry, horticulture, sheep and beef, and dairy land in FHZs. Temporal changes in exposure of agricultural land are reported at regional levels, and changing flood exposure is described in the context of shifting economic conditions. Finally, we discuss the importance of monitoring and investigating land-use change in floodplains to inform risk management under changing climatic and socio-economic conditions.

## 2. Agriculture and Flood Hazards

### 2.1. Regional Setting

A-NZ is a diverse but mostly oceanic, temperate environment with a sub-tropical region in the North and cooler temperate areas in the South [29]. Agricultural activity takes place throughout the country and has increased in diversity as irrigation and cultivation systems have become commonplace [30]. Primary production land covers 6.6 million hectares in the North Island and 6.5 million hectares in the South (58% and 43% of the total land, respectively) [31]. Agriculture has long been a vital component of A-NZ’s economy, both internally and in global export markets. In 2018, agriculture made up 7% of the Gross Domestic Product (GDP), with NZD 12.4 billion dollars of value added. In 1990, this was 8% and nearly NZD 4 billion in value [32]. The A-NZ economy is highly reliant on international trading, with agricultural and forestry products making up 79% of total exports [33].

Flood risk in A-NZ is driven by several factors that influence both the hazard intensity, such as high-intensity rainfall occurrence, catchment conditions, and climate change, and societal risk, such as increased development and the vulnerability levels of populations and industries within floodplains [1]. The fertile soils and gentle topography of floodplains provide an ideal setting for agricultural land types, making farms highly exposed to flood hazards [9].

### 2.2. Flood Hazard Mapping

In A-NZ, the quantification of flood hazard is primarily undertaken by local regional, unitary, and territorial authorities to inform land-use planning, flood scheme design, and stormwater management [1]. The Ministry for the Environment (MfE) advises on this work at a national level through the publication of guidelines and policy briefs (Environment Act 1986, s31 (c) (iv)). Currently, flood hazard is calculated and represented in numerous ways across the different regions dependent on the available data (e.g., LiDAR quality), the amount of funding available, and historic incidence, and approaches to flooding. This results in several inconsistences between datasets, such as differences in event severity and resolution used, thus creating challenges when compiling national flood maps.

## 3. Methods

### 3.1. National Flood Hazard Zone Map

**Figure 1.**
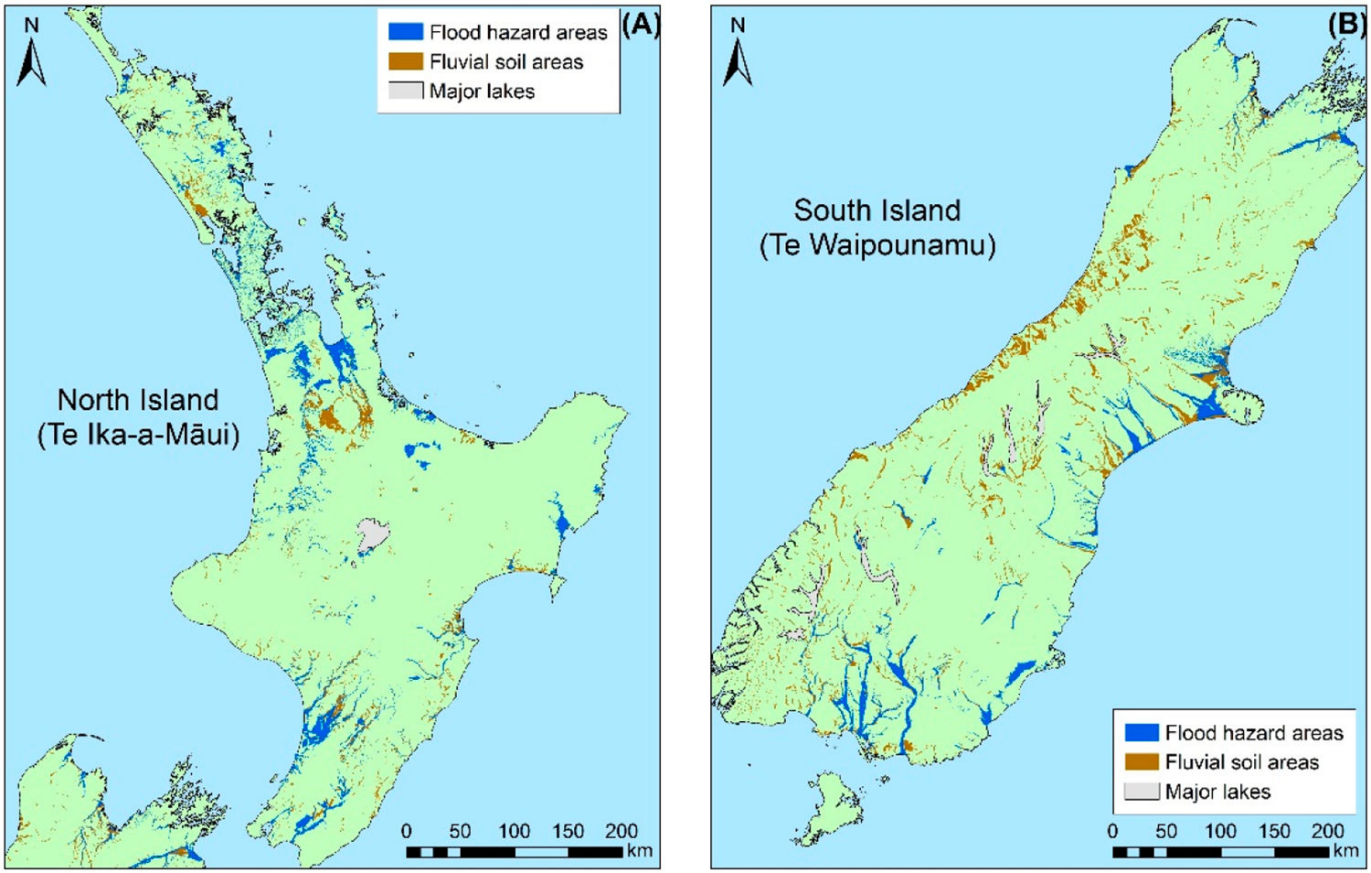
Mapped (ArcGIS Pro) flood hazard areas and FSL fluvial soil areas for (**A**) the North Island and (**B**) the South Island of A-NZ (for FHZ map sources, see Supplementary Material S1; fluvial soil areas were sourced from LRIS Portal [35]).

Maps of floodplains were principally collected from local authorities, such as regional councils (initial collection in August 2018, updated in December 2020). These are accessible for use under the Creative Commons License (New Zealand). Maps were available for all 16 regional/unitary areas, with coverage and methodologies varying significantly (Figure 1, Supplementary Material S1). The methodologies used included: 2D numeric modeling with LiDAR input, 1D with river cross-sections, GIS mapping using LiDAR and previous flood event levels, and digitized historic flood field mapping and aerial photographs. This led to varying event magnitudes and frequencies and associated levels of uncertainty being modeled across regions. Mapped FHZs usually represent 0.5%, 1%, 2%, or 5% AEP inundation scenarios and do not commonly account for any residual risk.

A further limitation of these publicly available datasets is their focus on urban areas and locations with highly concentrated infrastructure, rather than areas with agricultural investments. Extending the FHZ map for agricultural land required the inclusion of the ‘Flood Soil Layer (FSL)’ national dataset, where no flood maps were available. The FSL dataset is divided into six fluvial soil classes, with five of these assigned an estimated flood frequency based on flood scheme reports, soil types, and expert opinion [34]. A geospatial accuracy assessment comparing urban areas (Auckland, Hamilton, Wellington, Christchurch, and Dunedin) covered by publicly available flood hazard datasets with the FSL data demonstrated a greater than 75% agreement in the identified FHZ area (using the ‘summarize within’ tool in ArcGIS Pro). Therefore, the FSL data were used to indicate potential flood hazard in areas where modeled and historic flood hazard maps were not available (primarily rural areas) (Figure 1).

The modeled, historic, and publicly available FSL vector datasets were combined to create a composite FHZ polygon for A-NZ. Ideally, a series of nationally consistent modeled FHZ maps for representing numerous AEP intervals would be presented and exposure analyses would be undertaken for each scenario; however, this information is not currently available at a national scale. Therefore, this study applies an amalgamation of different flood magnitudes and frequencies that are used by government authorities to indicate areas at risk of flooding.

### 3.2. Land-Use and Carbon Analysis System (LUCAS) Mapping

We used Land-Use and Carbon Analysis System (LUCAS) mapping to assess agricultural land-use change in A-NZ. LUCAS tracks land-use change for the purposes of reporting A-NZ’s greenhouse gas emissions [36]. Land-use has been mapped since 1990, with datasets for 1990, 2008, 2012, and 2016. The mapping was compiled using Landsat (4, 5, 7) and SPOT (5) national satellite imagery, with MODIS, SPOT (2, 3), DMC, and aerial photography used to supplement the imagery [37]. Land use is categorized into 12 main classes and 30 subclasses for the 2008, 2012, and 2016 datasets, with no subclass information available for the 1990 data. These were assigned to key agricultural industries for this study (Table 1).

In order to give the LUCAS data an economic qualifier, earnings before taxes and income (EBIT) for the years 2008, 2012, and 2016 were compiled for a range of agricultural practices from various sources [38-47]. These sources primarily relied on business demography statistics compiled by Statistics NZ to provide average EBIT values for given years and agricultural land uses. This dataset uses the national business register and associated economic indicators (e.g., export values) for agricultural industries [48]. To reflect inflation over time, the EBIT of agricultural land uses for 2008, 2012, and 2016 were adjusted with a Consumer Price Index (CPI) [49]. The CPI values have been successfully applied in widely used annual budgeting reports compiled by Lincoln University and used by A-NZ farmers and rural professionals when valuing agricultural assets [44-47]. Other methods, such as traditional Power Purchasing Parities [50,51] and the creation of supply and demand curves, were considered but ultimately not employed to contextualize the study’s results in a way that would allow agricultural personnel to draw comparisons with previous EBIT valuations that they may have undertaken.

A Consumer Price Index (CPI) ratio was applied to calculate the EBIT values for 1990 using the 2017 EBIT figures, as these were the most recent figures available. A CPI is a measure of the average change in prices that consumers will pay for a given product over time, which is primarily driven by inflation, and it can be represented by Equation (1) below [49]:

(1) $$CPI_{t}=\frac{C_{t}}{C_{0}}\times 100$$

where *CPI_t_* is the *CPI* in the current period, *C_t_* is the cost of the given product at the time, and *C*_0_ is the cost of the given product in the base period.

This was calculated using a ratio of 2017 EBIT figures to give the 1990 EBIT (Equation (2)), where NZD 1.00 of goods and services in 2017 would have cost NZD 0.59 in 1990 (2017 CPI value of 1006.0, 1990 *CPI* value of 596.3) [52].


(2) $$CPI_{1990}=\frac{C_{1990}}{C_{2017}}\times 100=\frac{596.3}{1006.0}\times 100=\$0.59$$

These were then matched to the LUCAS land-use types to give a value in New Zealand Dollars (NZD) per hectare of agricultural land (Supplementary Material S2). EBIT values were then applied to agricultural land identified using the RiskScape exposure methodology (Section 3.3) to quantify the economic value of land exposed to the identified FHZs. Dairy, sheep, and beef EBIT values for 1990 are not given, as at this time, the LUCAS dataset did not include pastoral sub-types.

### 3.3. Exposure Analysis

Exposure mapping was undertaken by using the RiskScape multi-hazard risk model framework [53]. This modular framework is configured to produce exposure and impact data by analyzing hazard data and the characteristics of the assets at risk, and it has been applied in exposure studies in New Zealand and the wider Pacific region [54-58]. The RiskScape engine computes user-defined risk/exposure/vulnerability calculations for these hazards and assets.

**Figure 2.**
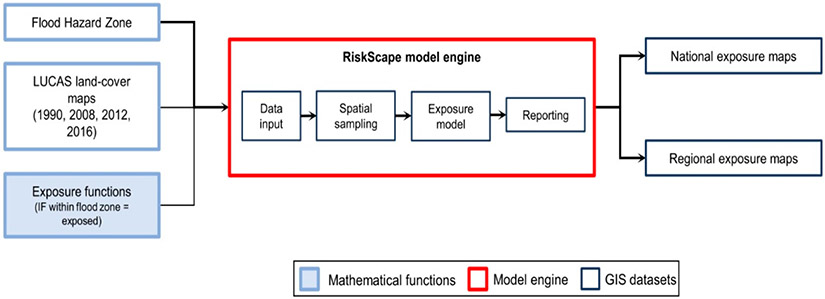
Workflow of the RiskScape model applied in this study to quantify agricultural land exposure in FHZs (based on [55]).

In this study, agricultural land exposure in A-NZ was quantified with respect to the national FHZ layer. FHZ and LUCAS data were used as input, along with a function providing RiskScape with the exposure calculation to apply. RiskScape geospatially aligns the vector layers representing the FHZ and LUCAS land-use maps to calculate the area extent (ha) and economic value (NZD) of each agricultural type exposed to flooding. The software engine then aggregates and reports agricultural land exposure nationally and by region (Figure 2).

## 4. Results

### 4.1. National Agricultural Land Exposure in FHZs

**Figure 3.**
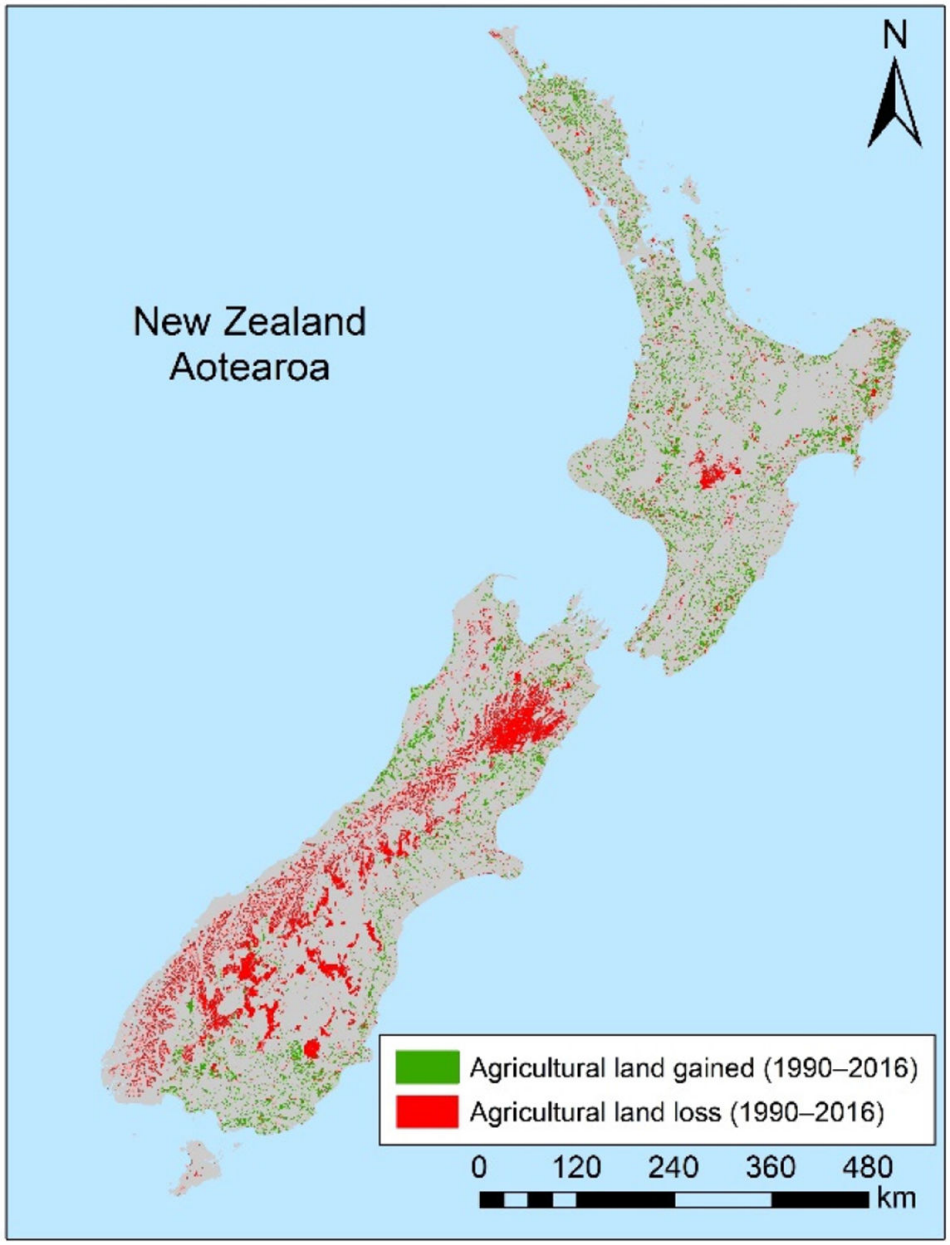
Agricultural land-use change in A-NZ 1990–2016 defined by the LUCAS dataset, showing areas that were not used for agriculture in 1990 but were in 2016 (agricultural land gained) and areas that were agricultural in 1990 but were no longer by 2016 (agricultural land lost) (areas sourced from [35]).

The agricultural land within A-NZ’s FHZs decreased slightly from 1.57 million to 1.5 million hectares between 1990 and 2016. This decrease occurred primarily in the South Island (57,000 ha decrease; Table 2; Figure 3).

Pastoral land within FHZs nationally decreased between 1990 and 2016 by 2.4% (~100,000 ha; Table 2). However, higher-value dairy farming comprised a greater percentage of this exposed land, especially in the South Island, where dairy farming within FHZs increased by 8.9% (~78,000 ha) between 2008 and 2016 (there was a 4.1% or ~28,000 ha increase over the same time period in the North Island). In contrast, the overall amount of arable, forestry, and horticultural land within FHZs remained relatively consistent with increases of less than 1% nationally between 1990 and 2016 (Table 2).

The potential annual EBIT for FHZ dairy farming increased by nearly NZD 890 million from 2008 and 2016. Conversely, the potential EBIT from sheep and beef farming, arable land, and forestry in FHZs decreased over this time (decreases of NZD 107 million, 10 million, and 4 million, respectively; Table 3). A sharp rise in the amount and value of dairy farming, along with a more gradual increase in horticultural land area and production value, drove an increase of NZD 1.9 billion in the total potential EBIT from agricultural land within FHZs between 1990 and 2016, thus substantially increasing the overall economic exposure of agricultural industries (Table 3).

### 4.2. Regional Exposure

**Figure 4.**
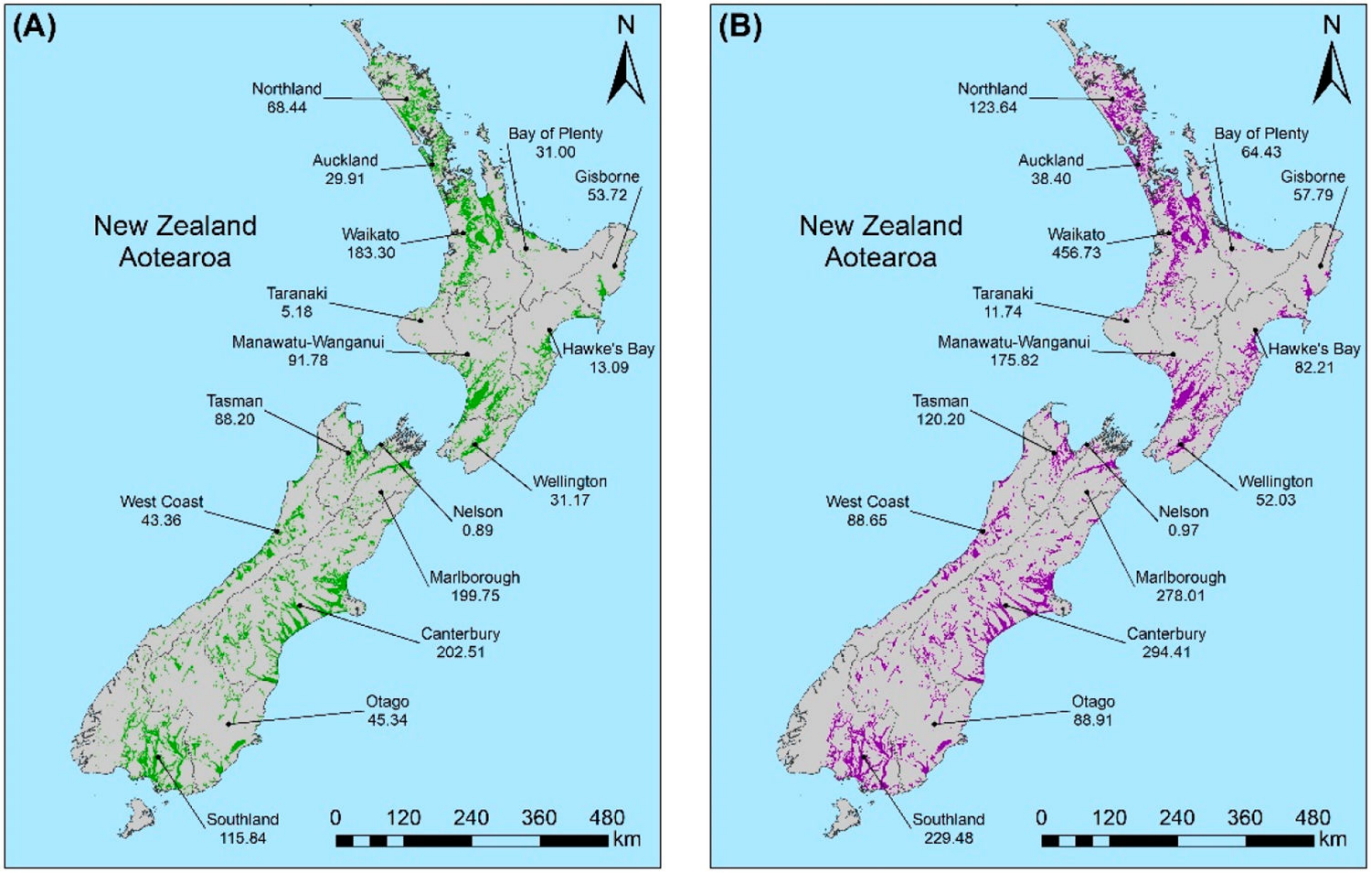
Map showing the regional exposure of agricultural land uses within FHZs and the associated EBIT values in (**A**) 2008 (in green) and (**B**) 2016 (in purple). N.B., 1990 values are not shown, as the division of pastoral land-use types is not available; therefore, accurate EBIT values could be calculated.

Most of the regional change in annual EBIT appeared to be driven by the rising price of agricultural products. This occurred in two ways: firstly, by causing land-use types that had a similar exposed area within FHZs throughout the study period to continue to increase in value due to the growth in annual EBIT per hectare, and secondly, by providing the impetus for farmers to convert to high-value dairy farming as the rise in its EBIT significantly outpaced that of other agricultural types (Figure 4).

**Figure 5.**
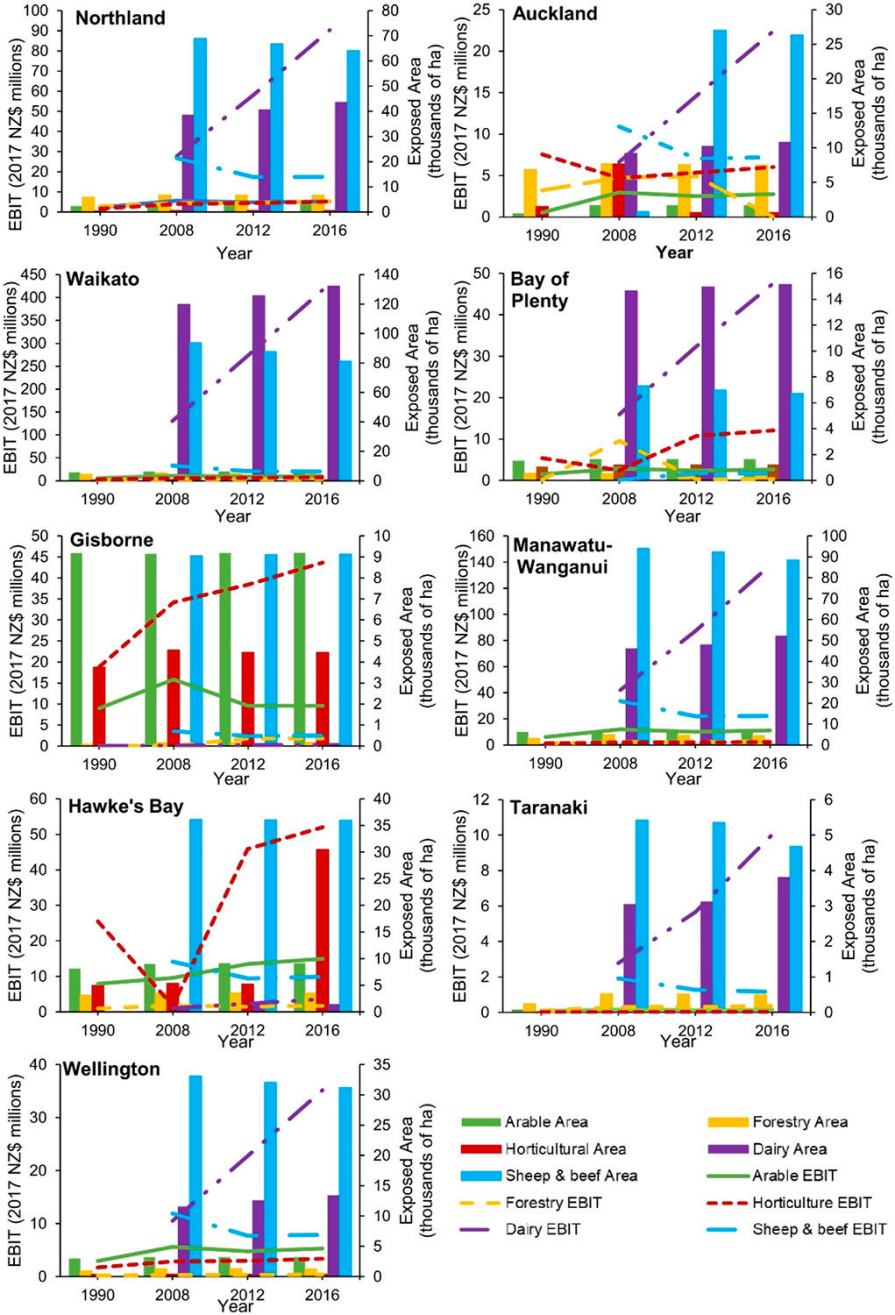
Regional changes in agricultural land area (hectares) and EBIT (2017 NZD million) within FHZs on the North Island between 1990 and 2016.

**Figure 6.**
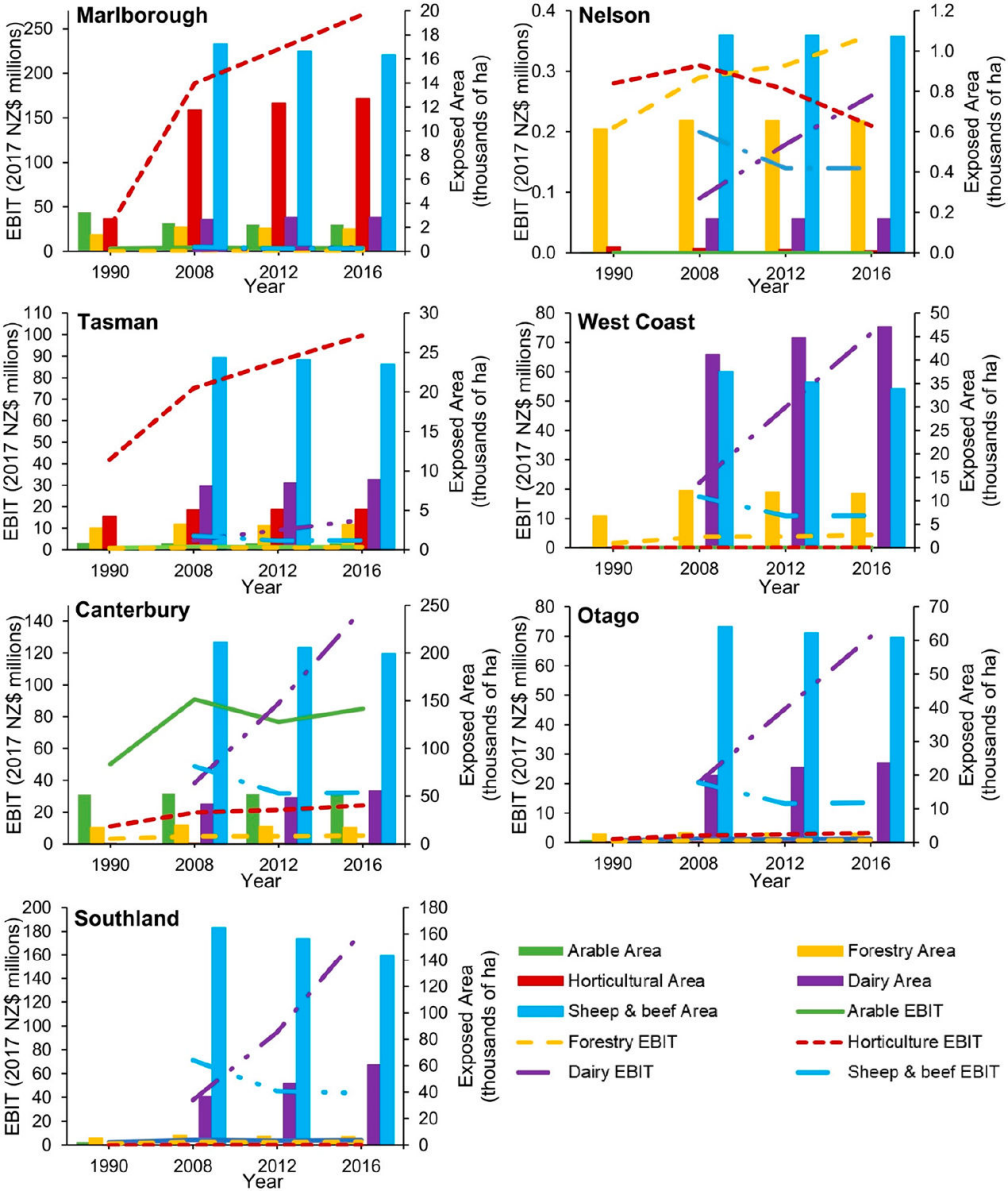
Regional changes in agricultural land area (hectares) and EBIT (2017 NZD million) within FHZs on the South Island between 1990 and 2016.

While national exposure remained relatively stable over the study period, regional variability in land and EBIT was observed for specific industries. Regionally, arable land area remained relatively stable between 1990 and 2016. In contrast, forestry showed an increase between 1990 and 2008, followed by a decrease between 2008 and 2016 for most regions (Figures 5 and 6). This reflects the increase in economic value and returns on dairy farming, thus incentivizing conversion away from forestry, rather than issues with forestry product profitability.

Horticultural land area remained consistent across most regions between 2008 and 2016. Marlborough and Hawke’s Bay, however, experienced notable increases in horticultural land (15,000 and >20,000 ha, respectively), whereas Auckland showed a substantial decrease from 1500 to 600 ha between 2008 and 2012. The land increases in Marlborough and Hawke’s Bay reflect investments in the viticulture industry, whilst the land decrease in Auckland was driven by urban expansion (Supplementary Material S3).

The Southland, Canterbury, and Waikato regions were where the greatest dairy expansion within FHZs took place (between 2008 and 2016; Figures 5 and 6; Supplementary Material S3). Most regions experienced dairy expansion as land was converted from the lower-value land-use types of forestry or sheep and beef farms. The conversion of other agricultural types to dairy farming was further evidenced by the decreasing sheep and beef farming being more evident in regions with a significant increase in dairy farming (i.e., Southland and Canterbury, Figure 5; Waikato and Manawatū-Wanganui, Figure 5).

Overall, Hawke’s Bay was the only region to show an increase (23,000 ha) in FHZ agricultural land within this time (Supplementary Material S3), which was primarily driven by horticultural expansion. This increase was likely an overestimation due to improvements in the identification of land-use types driven by remote sensing technology development; however, a significant increase in horticulture in the area has been reported elsewhere [59].

The largest increase in the annual EBIT of agricultural land within FHZs between 2008 and 2016 was in the Waikato region (NZD 273 million increase; Figure 5; Supplementary Material S4). This was due to the increase in the both the area of high-value dairy farming and its earning potential per hectare. Horticulture showed a more modest increase than dairy farming (NZD 335 million between 1990 and 2016), which was mostly driven by the increasing value and expansion of the horticulture land in the Hawke’s Bay (Figure 5), Marlborough, and Tasman (Figure 6) regions.

Forestry and arable annual EBIT in FHZs remained steady for most regions as the increase in value per hectare of forestry was absorbed by the decrease in forested area, whilst for arable land, the area and value of products were more consistent. The exception to this was in Nelson, where the arable area increased and the forested area remained the same.

Although there was an increase in annual EBIT per hectare of sheep and beef products between 2008 and 2016, the conversion of these farms to other land-use types (primarily dairy) drove a decrease in its EBIT for all regions (except for the Bay of Plenty, where sheep and beef land area remained the same; Figure 5).

## 5. Discussion

Exposure studies such as this are vital for understanding evolving risk profiles and the resulting vulnerabilities for important industries, such as agriculture. Agriculture is highly exposed and vulnerable to flooding. This is due to productive land often being located within floodplains, where fertile soils form, as well as its reliance on a complex and interconnected range of assets that are susceptible to damage from flooding, such as pastoral vegetation, crops, livestock, farming machinery, fencing structures, and external contractors and customers [26]. Additionally, the exposure and vulnerability of agriculture are increasing due to climate change, as there will be a non-linear increase in flood discharge levels as rainfall intensity increases with the rise of the average temperature [60]. Average temperatures in A-NZ are predicted to rise by 3 °C by 2090, which will lead to a 34% increase in the magnitude of a 1-in-10 year flood event over a one-hour duration (15% over a five-day period) [61]. The relative sea level has risen 2.44 mm per year in the last 60 years and is predicted to increase by an additional 0.67 m by 2090. This sea-level rise will increase the intensity of flood events in coastal areas; additionally, groundwater levels will also increase with sea-level rise, causing higher peak flood discharges, even relatively distal to the coast [62]. These factors will lead to more agricultural land being exposed to flooding more frequently and in more severe ways (i.e., greater depths and duration).

Additionally, this study shows that the agricultural land that remains within FHZs is growing more valuable as it is converted to dairy farming (nationally) and horticulture (Hawke’s Bay, Nelson, and Marlborough) from forestry and sheep or beef farming. Dairy farmland within floodplains increased substantially by 106,000 hectares between 2008 and 2016. This equates to an increase in potential EBIT in FHZs of over NZD 1 billion (in 2017 NZD). This is driven by an increase in the profit margins for dairy and horticulture between 1990 to the early 2000s causing landowners to accept more risk and expand these high-value operations into floodplains [63]. This trend is especially evident in Southland, Canterbury, and Waikato. In addition to increasing vulnerability with widespread dairy farming, the earning potential of the land has also increased substantially. This will lead to greater economic damage from flood events and more expensive insurance claims [1]. Similar trends in increasing vulnerability are being observed in Eastern Europe, where wetlands and crops are being converted to less resilient pastoral agriculture, including dairy farming [64].This is also occurring in Italy, India, and the UK, where economic reliance on dairy farming in floodplains continues despite a notable increase in flood frequency [17,65-67], and globally, where the diversification of agricultural systems required to be resilient to flooding under various climate change scenarios is not being uniformly undertaken due to economic and social constraints [68,69]. The widespread occurrence and, in some cases, expansion of dairy farms in floodplains is driven by high global demand and increasing prices for dairy products compared to other agricultural products (such as meat, wool, and crops).

An additional mechanism that further increases agricultural exposure is that the the change toward more labor-intensive land-use types, such as dairy farming and horticulture, compared to more ‘hands-off’ types, such as forestry and arable farming, results in a greater number of people and capital assets being exposed to flooding. In particular, dairy farming requires a higher number of employees (e.g., more staff for milking and interventions to promote milk yields in dairy farming compared to traditional cattle farming) and higher-value fixed assets (e.g., milking sheds, milk storage and refrigeration units on dairy farms compared to more simple feed shelters on cattle farms) [70,71]. This also likely transfers the vulnerability to the wider community, who relies on these farms for employment and purchasing auxiliary goods and services. These factors, along with our growing socio-economic reliance on the dairy industry, significantly increase social and economic vulnerability within FHZs and surrounding communities [66]. Further assessments should also consider how population growth and economic pressures are leading to urbanization of traditionally rural catchments and the influence that these movements will have on flood exposure levels.

The key requirement of undertaking this exposure study was access to the FHZs and to the LUCAS land-use dataset. Internationally and in A-NZ, these datasets are becoming widely available to the public. Our spatio-temporal modelling framework relies on the accuracy of input datasets. Some of the most important limitations of this study were the differences in methodologies used in the various FHZ maps compiled. These included a range of numeric models used, AEPs represented, model resolutions, and approaches to incorporating the effects of climate change. Additionally, as the LUCAS land-use dataset was compiled from satellite imagery over several years, the methodology and image processing applied have vastly approved. This underlines a key issue with the datasets: Whereas data from multiple years are compiled (for FHZ data) and compared (for land-use data), they are often representative of a historical process that introduces assumptions into the spatio-temporal exposure assessment. Another methodological limitation of this study is the inaccuracy in the LUCAS land-use dataset, which leads to agricultural land being incorrectly classified. It is assumed that these issues will not significantly influence results when reported at regional and national scales.

Flood hazard mapping is generally undertaken at a local scale to inform urban flood management, with agricultural areas generally being of lower priority. However, the resourcing and expertise available for this task are not uniform across the country. There are significant inconsistencies in access to and quality of LiDAR and the level of modeling undertaken, from the mapping of historic events to fully probabilistic hazard models that incorporate climate change impacts. When considering agriculture, these inequities are magnified, with many authorities not being resourced to map FHZs outside of urban areas. This results in the inability to determine the frequency of exposure for agricultural land, which could be addressed through national-scale mapping initiatives. Internationally, there is evidence that the level of exposure and vulnerability of populations can be altered dependent on the scale at which analyses are conducted, demonstrating the importance of consistency in modeling [72].

Whilst agricultural land-use types are generally preferrable to built settlements in terms of reducing population exposure within FHZs, increasing the value of assets within FHZ is also not aligned with current flood management goals. Dairy conversions require different consenting and planning permissions depending on which regional or unitary council area the land is in [73]. Whilst there are currently no specific considerations of natural hazards when converting agricultural land-use types, consideration of potentially causing an increase in risk levels (either through greater exposure or increasing vulnerability) should be incorporated into conversion decisions with respect to the Resource Management Act (1991) and Building Act (2004). Policymakers should assess risks to different land-use types to inform appropriate growth in and around floodplains. To better understand the variable impacts that flooding will have on agricultural systems and inform land-use policy decisions, quantitative impact and risk assessments are required [26]. This information allows for spatially accurate, specific mitigation measures to be employed to minimize agricultural damages. Applying the exposure methodology presented here, along with vulnerability models, will allow for farm-scale risk assessments that can quantify direct and indirect agricultural impacts to be undertaken in the future. This would require high-resolution data on the likelihood of a given hazard intensity occurring (e.g., flood depth, flow velocity, etc.), as well as the quantitative evaluation of specific farm-scale vulnerabilities by considering individual farm characteristics and interdependencies [74-76]. To facilitate this analysis, an improvement in the amount and resolution of farm-scale spatial data is required, including further production land classes and more information on the capital asset locations and types that comprise farm systems. Current data availability means that it is not possible to perform these detailed quantitative impact assessments; however, improvements to hazard, land-use, impact, and vulnerability datasets that will allow this are ongoing. These future farm-scale analyses are important, as they can provide specific information that can inform rural land-use planning, emergency management, and mitigative strategies, especially in the face of ongoing climate change and increasing farm-system vulnerability.

## 6. Conclusions

This study presents the first spatio-temporal flood exposure assessment for agriculture in A-NZ. Agriculture is vitally important to the social and economic well-being of A-NZ, but it is highly exposed to frequent flood hazards. Understanding how exposure is evolving with land-use change informs future risk assessments and emergency management planning. This study also demonstrates a methodology that can be subsequently applied as updated FHZ and land-use maps become available. This is especially important in the context of climate change and fluctuating socio-economic pressures that cause changes in exposure patterns.

The amount of dairy farming and horticulture within FHZs in A-NZ increased between 1990 and 2016, which was primarily driven by the increasing profitability in these agricultural industries. This has caused an increase in vulnerability, as these types of farming are more reliant on fixed assets (such as vulnerable vegetation, farm machinery and buildings, etc.) that are likely to be damaged in a flood, in addition to requiring a larger number of workers that will be potentially exposed to flooding. Additionally, the assets and land exposed are of a higher value when converted to dairy farming, thus placing further pressure on insurance companies and regional economies after an event.

Further work is needed to be able to undertake detailed vulnerability and impact assessments both nationally and at a farm scale in order to quantify the effects of the increasing vulnerability caused by land-use change trends within FHZs. This is especially important when considered in the context of climate change and the resulting increase in flood event occurrence and severity, growing population exposure due to urbanization, and the surge in the value of agricultural assets within FHZs.

## Supplementary Material

Flood map metadata and sources

## Figures and Tables

**Table 1.** LUCAS agricultural land-use classes used in this study [34].

LUCAS Land-Use Classes		LUCAS Land-Use Subclasses	Agricultural Land-Use Class
Forest land	Pre-1990 planted forest	Unknown; Pinus radiata; Douglas fir; Unspecified exotic species	Forestry
	Post-1989 forest	Unknown; Pinus radiata; Douglas fir; Unspecified exotic species	
Grassland	High producing	Unknown; Grazed dairy	Dairy
		Grazed non-dairy	Sheep and Beef
	Low producing	Unknown; Grazed non-dairy	Sheep and Beef
		Grazed dairy	Dairy
Cropland	Perennial	Unknown	Horticulture
	Annual	Unknown	Arable

**Table 2.** Changes in national agricultural industry land (hectares) exposure within FHZs between 1990 and 2016.

Area	Year	Arable	Forestry	Horticulture	Total Pastoral	Dairy	Sheep and Beef	Total
National	1990	95,699	61,445	20,878	1,396,083			1,574,104
	2008	103,463	67,372	32,396		364,351	891,211	1,458,792
	2012	99,979	75,257	32,193		429,278	864,307	1,501,014
	2016	99,991	72,810	32,524		470,579	825,800	1,501,705
North Island	1990	36,883	24,990	12,714	644,318			718,905
	2008	41,290	30,392	13,659		243,679	375,380	704,400
	2012	41,453	29,921	13,287		256,083	363,122	703,866
	2016	41,452	29,109	13,257		272,097	347,518	703,432
South Island	1990	58,816	36,455	8,163	751,765			855,199
	2008	62,174	36,980	18,737		120,672	515,831	754,393
	2012	58,526	45,336	18,906		173,195	501,185	797,148
	2016	58,539	43,701	19,267		198,482	478,282	798,272

**Table 3.** National EBIT for agricultural industries for 1990, 2008, 2012, and 2016 (in 2017 NZD).

Area	Year	Arable	Forestry	Horticulture	Dairy	Sheep and Beef	Total
National	1990	93.16	18.10	147.92			259.15
	2008	167.65	39.74	343.74	361.81	305.79	1218.73
	2012	143.21	32.17	418.78	783.41	198.71	1576.28
	2016	157.75	36.79	483.18	1251.26	198.23	2127.21
North Island	1990	35.91	10.43	64.75			111.09
	2008	65.45	26.28	57.48	237.28	136.35	522.84
	2012	57	18.81	79.48	494.92	89.9	740.11
	2016	62.12	21.49	89.82	763.01	90.14	1026.58
South Island	1990	57.26	7.63	83.17			148.06
	2008	102.2	13.46	286.26	124.53	169.44	695.89
	2012	86.21	13.36	339.3	288.49	108.81	836.17
	2016	95.63	15.3	393.36	488.25	108.09	1100.63

## Data Availability

Publicly available datasets were analyzed in this study. LUCAS land-cover data can be found here: https://data.mfe.govt.nz/layer/52375-lucas-nz-land-use-map-1990-2008-2012-2016-v008/ (accessed on 7 March 2021). Flood hazard data sources are listed in Supplementary Material S1.
